# How Japanese companion dog and cat owners’ degree of attachment relates to the attribution of emotions to their animals

**DOI:** 10.1371/journal.pone.0190781

**Published:** 2018-01-05

**Authors:** Bingtao Su, Naoko Koda, Pim Martens

**Affiliations:** 1 International Centre for Integrated Assessment and Sustainable Development (ICIS), Maastricht University, MD Maastricht, The Netherlands; 2 School of Agriculture, Tokyo University of Agriculture and Technology, Saiwai-cho, Fuchu, Tokyo, Japan; Leiden University, NETHERLANDS

## Abstract

Recently, studies in the United States and European countries have shown that the degree of attachment is associated with the attribution of emotions to companion animals. These studies imply that investigating the degree of attachment to companion animals is a good way for researchers to explore animal emotions and then improve animal welfare. Although a promising area of study, in Japan, no empirical studies have examined the correlation between the degree of attachment and the attribution of emotions to companion animals. In this research, we aimed to assess companion animal owners’ attribution of six primary (anger, joy, sadness, disgust, fear and surprise) and four secondary (shame, jealousy, disappointment and compassion) emotions to their dogs and cats, as well as how the degree of attachment related to such attribution of emotions from a Japanese cultural perspective. The “Pet Bonding Scale” (PBS), which is used to determine the level of bonding between humans and animals, was introduced to measure respondents’ degree of attachment to their companion animals. The results of a questionnaire (N = 546) distributed throughout Japan showed that respondents attributed a wide range of emotions to their animals. Companion animals’ primary emotions, compared to secondary emotions, were more commonly attributed by their owners. The attribution of compassion and jealousy was reported at a high level (73.1% and 56.2%, respectively), which was surprising as compassion and jealousy are generally defined as secondary emotions. All participants were highly attached to their companion animals, and this attachment was positively associated with the attribution of emotions (9/10) to companion animals (all *p* < 0.05). This study is one of the first to investigate animal emotions by analyzing the bonding between companion animals and owners in Japan, and it can therefore provide knowledge to increase Japanese people’s awareness of animal welfare.

## Introduction

Human attribution of companion animal emotions is commonly used in an attempt to improve animal welfare [1–3]. An important ethical issue in animal welfare appears precisely due to the opinions held by many people that most animals have emotional experiences. If animals experience disappointment and fear due to an inability to perform their natural behavior patterns or, more directly, due to animal cruelty, then this has moral importance and, in turn, may have a major influence on animal welfare [4, 5]. Therefore, a better understanding of animal emotions is an important step toward promoting optimum animal welfare. Emotion is the mental state expressed by animals, and it reflects animals’ psychological reality [6]. A direct attempt to explore animal emotions is to attribute their emotions from human perspectives. However, the question of whether animals experience the same range of emotions as humans has long been argued [4, 7, 8]. Emotions can be classified into primary (e.g., anger, joy, sadness, disgust, fear, surprise) and secondary (e.g., shame, jealousy, disappointment, compassion) ones [9–12]. Primary emotions are, more than any secondary emotions, accessible to observation [10]. A growing number of studies have revealed that primary emotions are experienced by both humans and animals [13, 14], while secondary emotions are unique to mature humans and perhaps other primates, at least as presently understood [15, 16].

Several approaches have been adopted to study animal emotions [4]. For instance, researchers may investigate the role of emotions in human beings and then examine whether the function is the same in humans and non-human animals [17], or whether the mechanisms underlying emotions are similar in humans and non-human animals [4, 17]. In this research, we aimed to study animal emotions in a new way, investigating whether companion animal owners can attribute emotions to their animals and how the degree of attachment influences such emotion attributions from a cultural perspective. During the last several decades, many scales have been designed and developed to assess people’s attachment to animals [18]. After comparison with other scales, the “Pet Bonding Scale” (PBS) [18] was introduced in the present study to measure companion animal owners’ degree of attachment and its correlation with the attribution of emotions to their animals in Japan. The concise design, simple language, specific purpose and explicit meaning of each statement of the PBS allow it to be more easily understood by respondents and enable us to arrive at a single aggregated outcome [18, 19]. Additionally, compared to the lay public, companion animal owners would be a better choice when approaching animal emotions because their direct experience in interacting with animals may allow them to better comprehend animals’ behavior associated with emotions [5, 20].

To date, some studies conducted in Western countries have reported that companion animal owners can attribute a wide range of emotions to their animals [19–22]. Female owners are more likely to attribute emotions to their animals than are male owners [19, 23], while companion animal owners are more likely to attribute emotions to their dogs than to their cats [19]. Nevertheless, no significant differences in such emotion attributions have been found between companion dogs and cats in China, where people have a relatively low awareness of animal welfare [23]. Therefore, it is plausible that the attribution of emotions to companion animals might differ between different countries with varying awareness of animal welfare. However, in addition to human demographics and the different awareness of animal welfare, other variables, such as the degree of attachment to animals, traditional culture and ideological condition, might also influence companion animal owners’ attribution of emotions to companion animals. We selected Japan as the representative of this study since Japanese people are open-minded to different cultures. They appreciate the Western values of human rights and freedom and, simultaneously, respect the traditional Confucian and Buddhist values of harmony and humble behavior [24].

The relationships between humans and animals in Japan are largely influenced by its mentality of collectivism, traditional culture of Animistic Shinto, Confucianism and Buddhism, as well as the Western values of human rights and freedom [25]. Japanese people are collectivistic and not concerned with foundations or universal laws. They understand animal emotions in terms of complex interactions between dispositions of animals and contextual factors, and they might find it relatively difficult to separate animal emotions from the situational context in which they occurred [26]. Therefore, the fundamental attribution error is much harder to demonstrate with Japanese people than Western people [27]. Regarding traditional Japanese culture, Shintoism advocates reciprocal care and compassionate relationships between humans and animals. In addition to Shinto ideology, Japanese attitudes toward animals and animal emotions have been influenced by Confucianism, which highlights the symbiosis between humans and animals, although humans are regarded as the lords of creation [28]. Animals are often portrayed as being appreciative of and dutiful to humans in Japanese folklore and fables [25, 29], which reflects the earlier attribution of emotions to animals in Japan. Buddhism, one of the most important religions, influences Japanese social values, including attitudes toward animals. Japanese mainstream Buddhist philosophers regard animals as sentient beings with the potential for better rebirth and salvation in the cycle of death and rebirth. The memorial service for dead companion animals is indicative of Japanese tradition, since premodern and many modern Japanese people believe animals have souls, emotions and feelings, even after their death [30].

The primary purpose of this research was to examine Japanese companion animal owners’ attribution of emotions to their dogs and cats. Additionally, we aimed to investigate how the degree of attachment influences the attribution of emotions to companion animals from Japanese cultural perspectives. Considering that many previous studies conducted in Western countries have demonstrated how human demographics and the communications between animals and owners influence the relationship between humans and animals [19, 31, 32], we assume such variables would also influence the degree of attachment to companion animals in Japan. Therefore, we examined the role of these variables in the relationship between companion animals and their owners.

## Methodology

### Ethics statement

This study was conducted using protocols approved by Maastricht University’s Ethical Review Committee Inner City faculties (ERCIC).

### Materials

Using the Emotions of Pets Questionnaire (S1 Questionnaire), we wanted to investigate how the degree of attachment of Japanese owners of companion dogs and cats relates to the attribution of emotions to their animals.

The questionnaire consisted of four sections. The first section covered demographic information including age, gender, educational level, companion animal species, animal protection/nature conservation/human health organization participation, the existence of a private garden, attitudes toward religion, and the main source of inspirations.

In the second section, respondents were asked to supply information about their companion animals’ basic characteristics (e.g., gender, size, age, neutered status and owners’ perceptions of their animals’ health condition), as well as their husbandry practices (e.g., how often do you brush your dog? Where does your pet sleep?). Additionally, respondents were asked if they were the main caregivers of their pets, whether they have other pets, how many years they have owned their pets, and why they have chosen to have pets.

The “Pet Bonding Scale” (PBS) [33], a 25-item Likert scale, was introduced in the third section. The PBS is a five-point scale ranging from 0 (strongly disagree) to 4 (strongly agree). The sum of the PBS scores indicates the degree of owners’ attachment to their companion animals, and a high score reflects a strong attachment. Examples of questions include “I like to spend a lot of time with my pet”; “I can tell secrets to my pet”; and “I keep pictures of my pet.” Furthermore, respondents were also asked how their pets communicated with them (e.g., meowing/barking, body language, touching, scratching, looking, sniffing) and how they communicated with their companion animals (e.g., watching, touching, petting).

In the fourth section, a list of six primary (anger, joy [happiness], fear, surprise, disgust and sadness) and four secondary (shame, jealousy, disappointment, compassion) emotions was given to the participants. They were asked whether they had witnessed any (or all) of these emotions expressed by their companion animals. Ratings were made on a three-point Likert scale (1 = never, 2 = sometimes, and 3 = often). A high score indicates a strong attribution of emotion to companion animals.

### Procedure

Data were collected from Japanese dog and/or cat owners using paper-based and online (n = 400) questionnaires (S1 Dataset). The paper questionnaires were conducted using the authors’ networks. By means of snowball sampling [34], 146 Japanese dog and cat owners filled in our questionnaire. The online questionnaire was conducted via Cross Marketing, one of the pioneer research companies in Japan, by means of simple random sampling [35]. The invitation email with the hyperlink to our questionnaire was sent to participants and they were asked to visit the website of our questionnaire and click “submit” when they complete all the questions. A total of 400 dog and cat owners were obtained from 1841 people throughout all the 47 prefectures of Japan. The response rates for the two survey methods were 100% and 21.7%, respectively. The inclusion criteria were as follows: 1) volunteers who were older than 18 years and who agreed to attend the study and 2) volunteers who were the main caregivers of their companion dogs/cats. To keep the answers consistent, participants were asked to respond for only one dog or cat. For those owners who owned more than one companion animal, we asked them to respond according to the animal they had owned the longest [19]. In the questionnaire, we explained the purpose of our study to the participants and stated that all information they provided would be kept completely confidential. Personal information would not be released to or viewed by anyone other than the researchers involved in this project.

### Statistical analysis

The correlations between respondents’ attribution of emotions and the degree of attachment to companion animals were analyzed with SPSS version 24 statistical software. Mann-Whitney U testing was performed to examine the different attribution of emotions between dogs and cats. Pearson correlation analysis was conducted to explore the relationships between the degree of attachment and the attribution of emotions to companion animals. Cocor, a software package, was utilized to determine the difference in correlations between dog and cat owners, as well as between male and female owners [36]. All results are based on two-sided tests, and values of *p* < 0.05 were considered significant. Considering that the PBS scores in this study followed a normal distribution, a stepwise linear regression was used to relate the degree of attachment (measured by PBS) to demographics and other basic information, such as animal welfare organization participation, the relationship with pets, how owners communicated with their pets (e.g., by watching, by taking care of pets) and how pets communicated with their owners (e.g., by looking, by touching). An alpha value of 0.05 was used for forwards and backwards regression of variables. To ensure that the observed correlations were not caused by autocorrelation, the Durbin-Watson statistic was used. Values of 2.0 were considered to have no autocorrelation, while values approaching 0 indicate positive autocorrelation and values approaching 4 indicated negative autocorrelation [37]. Stepwise regression was regarded as problematic because it could result in an inappropriate selection of predictors and the final model can vary according to the selection procedure chosen [38, 39]. Therefore, we only considered predictors appearing in the final model as influential variables in order to address these problems and simultaneously reduce type-I errors [40]. Additionally, using this method may also increase type-II errors, but given the relatively large sample size in the present study, this risk should be reduced [41].

## Results

### Human demographics

In total, 546 completed surveys (50.5% from men, 49.5% from women) were received. The mean (±) age of all participants was 48.66 (± 13.87) years. Companion animals’ basic information is reflected in Table 1. We compared our data from the two survey methods (i.e., paper-based and online questionnaires) in the present study, and the results showed no significant difference in the final results except that the participants from the paper-based questionnaire (*M* = 77.53) had slightly higher PBS scores than participants from the online questionnaire (M = 73.57, p = 0.01). Therefore, we combined the data from the two surveys in the following analyses.

**Table 1:** Companion animals’ basic information.

	Dog: N (%)	Cat: N (%)
Animal species	344 (63.0)	202 (37.0)
Gender		
Male	198 (58.4)	90 (44.6)
Female	141 (41.6)	111 (55.0)
Missing data	5 (1.5)	1 (0.5)
Age		
< 5 years	73 (21.2)	64 (31.7)
5–10 years	151 (43.9)	66 (32.7)
> 10 years	120 (34.9)	72 (35.6)

### The attribution of emotions to companion animals

**Fig 1:**
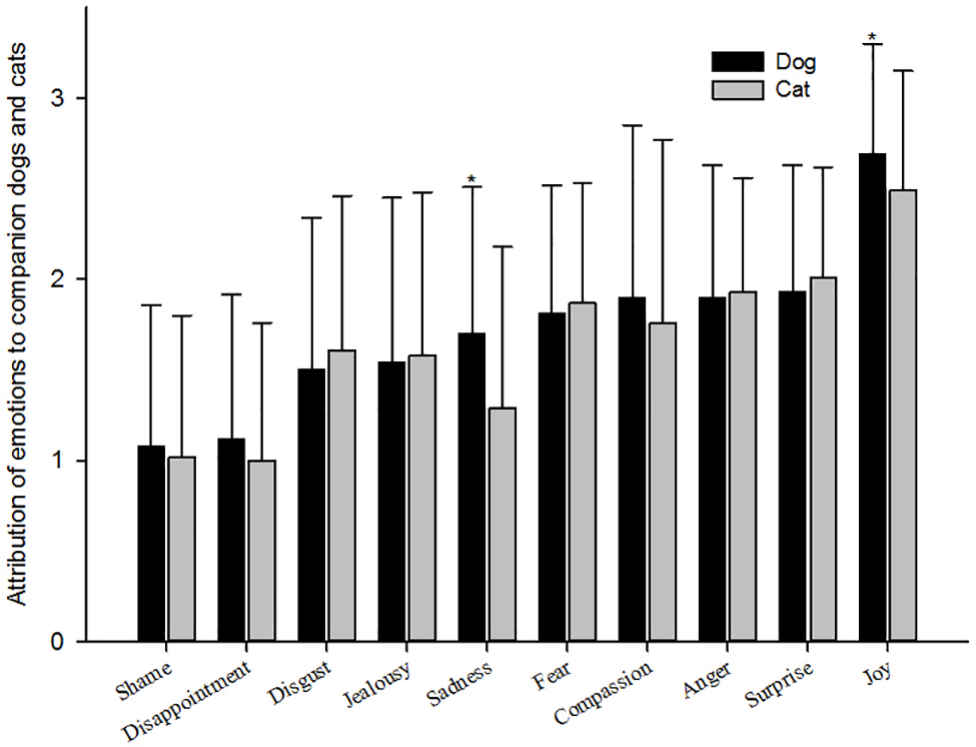
Attribution of emotions to companion dogs and cats. Note: An asterisk indicates significance of the attribution of emotions (joy [*p* < 0.001] and sadness [*p* < 0.001]) between companion dogs and cats; df = 544.

More than half of the respondents reported that they could often or sometimes attribute primary emotions of joy (96.2%), surprise (85.9%), anger (80.6%), fear (75.7%), sadness (61.9%) and disgust (57.7%) and secondary emotions of compassion (73.1%) and jealousy (56.2%) to their companion animals. According to the Mann-Whitney U test, emotions of joy and sadness were more frequently attributed to dogs than to cats (Fig 1). Our results also showed that female owners were more likely to attribute emotions of anger, joy, disgust, fear, surprise, jealousy and disappointment to their companion animals than male owners were (not presented in the figure).

### The degree of attachment to companion animals and its predictor variables

Our results showed that the Cronbach’s alpha value for the PBS in the present study was 0.958. The mean attachment (PBS) score of all the respondents was 74.63 (*SD* = 14.94) out of 100. Female owners showed a higher attachment score to their animals (*M* = 79.04, *SD* = 13.37) than did male owners (*M* = 70.13, *SD* = 15.14, *z* = -7.04, *p* < 0.001). Dog owners showed a higher attachment score to their dogs (*M* = 75.64, *SD* = 14.94) than did cat owners to their cats (*M* = 72.91, *SD* = 14.82, *z* = -2.25, *p* = 0.024).

#### Companion dogs

We considered all the possible demographics variables and the interaction paths between companion dogs and their owners that might associate with the PBS score. The Durbin-Watson statistic suggested no autocorrelation (*d* = 2.13). According to stepwise multiple regression analysis results, respondents who considered their relationship with their dogs to be “good” had a higher PBS score than those who felt they had a bad relationship. Female respondents had a higher PBS score than male respondents. Respondents who liked watching their dogs and who brushed their dogs frequently had a higher PBS score than those who did not. Companion animals’ numbers and their living places also influenced their owners’ PBS score: those respondents who owned other pets had a higher PBS score than those who only had one dog, while respondents whose dogs slept in their bedroom had a higher PBS score than those whose dogs slept in other places (e.g., kitchen, living room, garage and basement). In addition, the results also showed that those reporting that their dogs can stay at home alone scored higher on PBS than those who thought their dogs could not stay at home alone (Table 2).

**Table 2:** Predictors of scores on the Pet Bonding Scale (PBS) for companion dogs.

Y: PBS for dogs (*df* = 343)	Unstandardized Coefficients		Standardized Coefficients	Zero-order coefficients	*t*	*p*
	*B*	Std. Error	Beta			
(Constant)	97.72	9.66			10.11	< 0.001
X_1_: Your relationship with your pet: bad (1)–good (2)	11.15	1.56	0.36	-0.074	7.13	< 0.001
X_2_: Gender of owner: female (1)–male (2)	-6.20	1.42	-0.22	-0.297^**^	-4.36	< 0.001
X_3_: You like watching your pet: yes (0)–no (1)	-21.89	6.97	-0.16	-0.268^**^	-3.14	0.002
X_4_: You have other pets: yes (1)–no (2)	-4.92	1.66	-0.15	-0.203^**^	-2.97	0.003
X_5_: The frequency of brushing dogs: once or more times each day (1)–once or more times each month (2)	-2.21	0.92	-0.12	-0.181^**^	-2.41	0.016
X_6_: Your dog can stay at home alone: yes (1)–no (2)	-7.45	3.15	-0.12	-0.127^*^	-2.36	0.019
X_7_: Your pet sleeps in bedroom: no (0)–yes (1)	1.84	0.88	0.10	0.172^**^	2.08	0.038

Note: Unstandardized and standardized coefficients refer to the partial effect of one predictor after adjusting for the others.

Zero-order correlation test

** *p* < .01.

* *p* < .05.

#### Companion cats

We also identified several predictor variables on the PBS score from the information we collected from cat owners. The Durbin-Watson statistic suggested no autocorrelation (*d* = 2.11). The results showed that respondents who considered their relationship with their cat to be “good” and who owned their cat for themselves showed a higher PBS score than those who considered their relationship with their cat to be “bad” and those who owned their cat for work or for children. Respondents who thought their behavior resembled their pet’s behavior had a higher PBS score than those who did not. Female respondents had a higher PBS score than male respondents. The PBS scores of respondents who had belonged to an organization involved in improving animal welfare were higher than the scores of those who did not. Furthermore, the interactions between owners and animals were also associated with the degree of attachment to cats: the PBS scores of respondents who liked taking care of their pets and who thought their pets communicate with them by touching were higher than the scores of those who did not. We also found that owners who had a garden had a higher PBS score than those who did not (Table 3).

**Table 3:** Predictors of scores on the Pet Bonding Scale (PBS) for companion cats.

Y: PBS for cats (*df* = 201)	Unstandardized Coefficients		Standardized Coefficients	Zero-order coefficients	*t*	*p*
	*B*	Std. Error	Beta			
(Constant)	67.70	9.57			7.08	< 0.001
X_1_: Your relationship with your pet: bad (1)–good (2)	7.06	1.72	0.23	0.449^**^	4.10	< 0.001
X_2_: Getting the pet for yourself: no (0)–yes (1)	6.96	1.66	0.23	0.433^**^	4.20	< 0.001
X_3_: Your behavior resembles your pet’s behavior: no (0)–yes (1)	5.16	1.75	0.16	0.313^**^	2.95	0.004
X_4_: You like taking care of your pet: yes (1)–no (2)	-9.13	2.77	-0.19	-0.428^**^	-3.29	0.001
X_5_: Gender of owner: female (1)–male (2)	-4.68	1.57	-0.16	-0.298^**^	-2.98	0.003
X_6_: Animal welfare organization participation: yes (0)–no (1)	-8.33	2.77	-0.16	-0.244^**^	-3.01	0.003
X_7_: Your pet communicates with you by touching you: no (0)–yes (1)	3.39	1.32	0.14	0.354^**^	2.56	0.011
X_8_: You have a garden: no (0)–yes (1)	3.92	1.56	0.13	0.162^**^	2.52	0.013

Note: Unstandardized and standardized coefficients refer to the partial effect of one predictor after adjusting for the others.

Zero-order correlation test

** *p* < .01.

### The correlation between the degree of attachment and the attribution of emotions to companion animals

Significant correlations were found between the degree of attachment (according to PBS score) and the attribution of primary (*r* = 0.262) and secondary emotions (*r* = 0.317, both *p* < 0.001). Specifically, there was a significant correlation between the degree of attachment and the attribution of joy, sadness, disgust, fear, surprise, shame, jealousy, disappointment and compassion to companion animals (all *p* < 0.01). Our results also showed significant correlations between the degree of attachment and female owners’ attribution of joy, disgust and compassion. Regarding male owners, this correlation was significant for five of the six primary emotions (with the exception of anger) and all four secondary emotions (all *p* < 0.05). The correlation between dog owners’ degree of attachment and their attribution of five of the six primary emotions (with the exception of anger) and three of the four secondary emotions (with the exception of disappointment) was found to be significant, while for cat owners, this correlation was significant for four of the six primary emotions (with the exceptions of anger and fear) and all four secondary emotions (Table 4). According to the results of cocor, we also found that the correlations between male respondents’ degree of attachment and the attribution of sadness, jealousy and compassion to companion animals were stronger than those of female respondents (all *p* < 0.05), and the correlation between cat owners’ degree of attachment and their attribution of joy was stronger than that of dog owners (*p* < 0.05).

**Table 4:** Correlation coefficients of PBS score and the attribution of emotions to companion animals.

	Emotions				
	Overall correlation	Female owners	Male owners	Dog owners	Cat owners
Anger	0.049	0.005	0.030	0.037	0.079
Joy	0.301**	0.200**	0.298**	0.224**	0.402**
Sadness	0.193**	0.091	0.262**	0.153**	0.218**
Disgust	0.171**	0.124*	0.158**	0.173**	0.186**
Fear	0.135**	0.019	0.139*	0.144**	0.131
Surprise	0.198**	0.066	0.217**	0.204**	0.205**
Shame	0.185**	0.100	0.244**	0.137*	0.263**
Jealousy	0.231**	0.100	0.300**	0.209**	0.279**
Disappointment	0.150**	0.066	0.199**	0.087	0.248**
Compassion	0.369**	0.229**	0.431**	0.327**	0.428**

Note: Pearson correlation test

** p < .01.

* p < .05.

## Discussion

The aim of this study was to investigate Japanese companion animal owners’ attribution of emotions to their dogs and cats, as well as how their degree of attachment relates to the attribution of emotions to their animals. The results indicate that respondents attributed a wide range of emotions to their companion animals, with women attributing more emotions than men. Dog owners showed a higher level of attachment to their dogs than cat owners to their cats, while female owners showed a higher level of attachment to their companion animals than did their male equivalents. The degree of attachment was significantly correlated with Japanese respondents’ attribution of five of six the primary emotions and all four secondary emotions to their companion animals, with the higher the level of attachment, the stronger the attribution of emotions to animals.

### Emotions attributed and species differences

Our findings indicate that companion animal owners more commonly attributed primary emotions than secondary emotions to their animals. This finding is in line with the results of earlier studies that reported a trend in which primary emotions were more commonly attributed to companion animals than were secondary emotions [19–21, 23]. However, the secondary emotions of jealousy and compassion, two exceptions to this finding, were frequently attributed to companion animals in Japan. The result of jealousy parallels earlier findings in Western countries, while the result of compassion is inconsistent with findings from Western countries [19, 20] but is in line with findings from China [23]. Compassion is a necessary condition for actions that are hardly ethically neutral, and it is more easily aroused among identified situations than among unidentified situations [42]. Japanese and Chinese people are more collectivistic than Western population. Their mentality is holistic, focusing attention on the contextual situation in which animal emotions are occurred and ascribing causality by reference to the relationship between animal emotions and the contextual factors [26, 43]. Therefore, we suppose the relatively higher probability of animal cruelty in China and Japan, compared to that in Western countries, would promote owners’ empathic abilities of compassion, which may affect their attribution of compassion to animals. Additionally, in Japanese and Chinese culture, the feeling of compassion reflects the principle of benevolence, one of the five basic elements of Confucianism [44]. Dogs and cats are regarded as sentient beings and as having the nature of compassion to all misfortunes [30, 44]. Japanese and Chinese people therefore tend to give more anthropomorphic descriptions of animal emotions than Western population. Another reason to explain companion animal owners’ similar attribution of compassion in China and Japan is their relativistic ideology. Animals in Japan and China have been respected as an essential part of human society. Nevertheless, due to the concept of special omens (e.g., a hen pheasant was seen as a good omen), they were commonly used in ceremonies, including sacrificial offerings of various sorts [28, 44]. Therefore, Chinese and Japanese people’s attitudes toward animals, including the attribution of emotions, are based on situational analysis, while Western populations’ attitudes toward animals are formed by their universal principle of animal welfare [45]. Notably, the attribution of emotions (particularly secondary emotions) to companion animals is complicated, and no one simple reason or theory can explain all of the psychological phenomena that are called “emotions” and “attribution of emotions”. Further evidence from neuroscientific or psychological perspectives is therefore needed to confirm and clarify this point.

Previous studies have demonstrated that people’s attribution of emotions to animals changes significantly depending on the different animal species, such as dog and cat [19, 46]. Our results also reveal this difference between dogs and cats, yet this difference was only reflected in the emotions of joy and sadness, which confirms that dogs are more expressive in their body language and facial expressions than cats are, especially when they feel joy and sadness [19, 46]. However, the lower number of emotional attributions implies that the relationship between companion animals and owners in Japan is different from that in Western countries. Many Japanese companion animal owners relate to their animals emotionally and with little knowledge about animal characteristics, such as their habit and behavior [47, 48]. Therefore, it is not surprising that Japanese companion animal owners can attribute emotions to their animals, but their attribution of emotions to companion animals was not as significantly different as that of Western populations regarding animal species. Women were found to be more frequently to attribute anger, joy, disgust, fear, surprise, jealousy and compassion to their companion animals than men were. This finding confirms previous surveys conducted in China and Australia reporting that women, compared to men, were more willing to attribute emotions to companion animals [23, 49].

### Predictor variables of the PBS

In addition to the attribution of emotions, we were also interested in the variables that predicted companion animal owners’ degree of attachment to their animals, since determining these variables is an important way to improve human-animal relationships and animal welfare. Our results reveal that there is general consent on considering the mutual interactions between animals and owners (e.g., for owners: watching and taking care of their pets; for pets: interacting with their owners by touching them) as rewarding experiences that can improve owners’ degree of attachment to their animals. In opinion surveys on relationships with animals, gender is sometimes found to be a correlated factor [19, 23, 50]. Our results confirmed this finding by showing that women had a higher PBS score than men, suggesting that women are more concerned with animals and are likely to have a more positive relationship with animals. Another interesting finding is that the independence of dogs can promote the good relationship between dogs and their owners. This result is in accordance with a previous finding on the relationship between livestock and owners [51]. We interpreted this result as likely resulting from the lower degree of trouble they cause their owners.

### Owner attachment and attribution of emotions

Our analyses demonstrate that all respondents were highly attached to their companion animals, and the attachment levels positively correlated with the willingness to attribute emotions to companion animals. This finding implies that a combination of animal experience (pet ownership) and strong attachment may promote owners’ brain activations to attributing emotions to animals [5]. The identification of this correlation could serve as an alternative to or complementary part of existing methods to assess animal emotions, as well as animal welfare. The correlation between the attribution of emotions and the degree of attachment was significant for most of the animal emotions in Japan. This finding is in accordance with earlier observations in European countries [19] but is different from results reporting that attributions of only a few significant animal emotions exist among female and male owners, as well as dog and cat owners in China [23]. Therefore, it seems that the degree of attachment plays a more important role in predicting Japanese and European people’s attribution of certain emotions to companion animals than that of Chinese people.

In addition, our results reveal that the correlation between male respondents’ degree of attachment and their attribution of certain emotions (sadness, jealousy and compassion) to companion animals was stronger than that of female respondents. Indeed, female respondents tend to be more attached to their animals than male respondents. Nevertheless, according to our results, the attribution of these emotions generally agreed between female and male respondents (with only minor deviations for jealousy), suggesting that the degree of attachment may be more prominent when considering such correlations. These results also demonstrate that the differences in the attribution of these emotions between male and female respondents were not as strong as the difference in the degree of attachment between male and female respondents. Additionally, our results reveal that dog owners were more attached to their dogs than cat owners were to their cats, although a stronger correlation between the degree of attachment and the attribution of joy existed among cat owners. This finding confirms that the difference in the degree of attachment between dog and cat owners was not as strong as the difference in the attribution of joy between dog and cat owners in Japan.

### Limitations of this study

Although this study is innovative, as it attempts to investigate the relationship between the degree of attachment and the attribution of emotions to companion animals from a Japanese cultural perspective, it is appropriate that we acknowledge the limitations. We used both paper-based and online questionnaire surveys to collect data, which may make the findings inconsistent. However, many previous studies reported that findings obtained by web survey are consistent with findings obtained by traditional paper-based survey [52–54]. Our results also showed non-significant differences between the two surveys, except for the minor deviations of the PBS score. We think this minor difference may be due to the unbalanced distribution of participants from the two method surveys.

## Conclusion

In the present study, companion animal owners are reported to attribute a wide range of emotions to their animals, with a trend toward primary emotions being more frequently attributed than secondary emotions. Most owners of dogs and cats also attribute a restricted range of secondary emotions of compassion and jealousy to their animals at levels comparable with primary emotions. Japanese people relate to their animals emotionally. They regard both companion dogs and cats as equally important, and both of them are associated with the spirit world. Therefore, their attribution of eight out of ten emotions to companion animals was not significantly different between dogs and cats. These findings are different from studies in Western countries showing that companion animal owners were more likely to attribute emotions to companion dogs than cats. We suppose the more collectivist mentality in Japan would explain these different findings between Japan and Western countries. Japanese people understand animal emotions in terms of complex interactions between dispositions of animals and contextual factors, whereas Western populations often view animal emotions primarily as the direct unfolding of animal dispositions. Additionally, our results provided evidence that the correlation between the attribution of emotions and the degree of attachment was significant for more animal emotions by Japanese and Western owners than by Chinese owners, which means that the attribution of emotions was more associated with the degree of attachment in Japan and Western countries than in China. Animal emotions have been identified as a critical marker for animal welfare, and thus, investigating methods for approaching animal emotions and exploring the correlations between the degree of attachment and the attribution of emotions to animals is essential to understand animal feelings and promote optimal animal welfare worldwide. Professionals who are expected to advise on animal welfare and human-animal relationships should take this correlation into account.

## Supporting information

S1 Questionnaire The emotions of pets. (PDF)

S1 Dataset Animal emotions in Japan. (SAV)
