# Supplementary material for: How Japanese companion dog and cat owners’ degree of attachment relates to the attribution of emotions to their animals
Source: PLoS One. 2018 Jan 5;13(1):e0190781. doi: 10.1371/journal.pone.0190781 (PMC5755896; doi:10.1371/journal.pone.0190781)
Supplement: S1 Questionnaire — (PDF) [file pone.0190781.s001.pdf]

The Emotions of Pets

Scientists more or less agree that emotions in humans act as a “mental guide” and affect our behavior. Emotions like fear, sadness and joy tell us which situations are good for us and which are bad. As a result of an emotion people react to a certain situation and may adapt their behavior. Emotions are therefore important for our functioning. Research has identified six basic emotions: anger, happiness, sadness, disgust, fear and surprise.

From an evolutionary point of view there seem to be no reason to assume that this would work differently in animals. However, it is difficult to determine whether they experience emotions in the same way as humans. It is not always clear what an animal feels, and you can easily be wrong in judging the nature and strength of their feelings. Yet in everyday life emotions are regularly assigned to animals. We might therefore assume that animals ‘use’ their emotions to adjust their behavior to a certain situation. Emotions are important for their functioning.

Through this survey we want to gain insights into how pet owners assign specific emotions to their pets based on facial expression, body posture, and sounds (barking, meowing, growling, etc.). We also want to know how the emotions of the owner and pet synchronize, in other words, looking at whether your pet tunes his/her emotions to those of you (or vice versa, for example, being sad/happy or angry simultaneously).

We would like to ask you (preferably the main carer - i.e. the person that devotes most time to the pet (walking the dog, feeding the cat, etc.)) to complete this questionnaire regarding the behaviour of your cat and/or dog and their emotions, and if possible, to send us a picture of your pet expressing one of the emotions mentioned above. This should take no longer than about 10 minutes of your time. By participating, we hope you receive personal satisfaction that you were part of a study to improve the lives of animals, and you may understand your own pet a little more afterwards. Of course, all information you provide will be kept completely confidential. Personal information will not be released to or viewed by anyone other than the researchers involved in this project. Results of this study will not include your name or any other identifying characteristics - unless you give permission for that.

1. Personal details:

Please give us some information about you, your house and your family. This information is kept strictly confidential.

1. What is your birth-year? (e.g. 1968)
2. What is your sex? Female/Male
3. What is the highest level of schooling you have completed?
   a. No education
   b. Less than grade 12
   c. High school
   d. College or technical school
   e. University
   f. No answer
   g. Other
4. Do you belong or donate to an organization or charity involved in or concerned with:
   a. Improving the welfare of animals Yes/No
   b. Conservation of the natural environment Yes/No

    c. Improving human rights or health Yes/No
5. Is religion/spirituality important in your life? Yes/No /No answer
6. If your answer is ‘yes’, what is your main source of inspiration (Multiple answers possible)?
    a. Christianity
    b. Judaism
    c. Islam
    d. Buddhism
    e. Taoism
    f. Shintoism
    g. Other

2. Your pet and its emotions

7. Do you own a dog or cat?
   If you have more than one dog/cat, please fill in the questions for the pet you have longest.

   Mark only one option.
   a. Dog Skip to question 8.
   b. Cat Skip to question 24.

Dog

Answer these questions if you own a dog.

8. What is the breed of your dog? (leave blank if unknown)
   ____
9. What is the sex of your dog? Male/Female
10. Is your dog neutered (fixed)? This applies for both male and female dogs Yes/No
11. How big is your dog?
    a. Small (less than 10kg)
    b. Medium (10-25kg)
    c. Large (more than 25kg)
12. How old is your dog?
    a. Less than 5 years
    b. 5 to 10 years
    c. 10 years or over
13. How would you rate the health of your dog? Good/Fair/Bad
14. How often have you visited the vet on average per year with your dog?
    a. Never
    b. Once or twice per year
    c. More than twice per year
15. On average, how often do you go for a walk with your dog each day?
    a. Twice or less
    b. Between two and four times
    c. Over four times
16. About how many minutes per day do you go for a walk with your dog?
    a. Less than 1 hour
    b. Between 1-2 hours
    c. More than 2 hours
17. How often do you feed your dog each day?
    a. Once
    b. Twice
    c. More than twice
18. On average, how many grams of food per kg body weight do you give your dog each day?
    (e.g., if your dog weights about 10kg, and you give him 100 grams, the answer is 10 grams)
    a. Less than 10 grams per kg bodyweight
    b. Between 10 and 25 grams per kg bodyweight
    c. More than 25 grams per kg bodyweight
    d. No idea
19. On average, how often do you brush your dog?
    a. Once or more times each day
    b. Once or more times each week
    c. Once or more times each month
20. Is your dog friendly to strangers?
    a. Yes

b. No
c. Sometimes yes/sometimes no

21. Can your dog stay alone at home? Yes/No
22. Where does your dog sleep?
    (Multiple answers possible)
    Check all that apply
    a. Kitchen
    b. Living room
    c. Bedroom
    d. Garage/basement/barn
    e. Hall
    f. Other
23. Who is taking care of your dog when you are (temporarily) not around?
    (Multiple answers possible)
    Check all that apply
    a. Shelter or kennel
    b. Neighbours, friends or family
    c. Other

Skip to question 39.

Cat

Answer these questions if you own a cat.

24. What is the breed of your cat? (leave blank if unknown)
    ____
25. What is the sex of your cat? Male/Female
26. Is your cat neutered (fixed)? This applies for both male and female cats Yes/No
27. How old is your cat?
    a. Less than 5 years
    b. Between 5 and 10 years
    c. 10 years or over
28. How would you rate the health of your cat? Good/Fair/Bad
29. How often have you visited the vet on average per year with your cat?
    a. Never
    b. Once or twice per year
    c. More than twice per year
30. How often does your cat go outside?
    a. Never
    b. Can go outside whole day
    c. Can go outside during daytime
    d. Can go outside during night time
    e. Only goes outside when someone is at home
31. How often do you feed your cat each day?
    a. Once
    b. Twice
    c. More than twice
32. On average, how many grams of food do you give your cat each day?
    a. Less than 50 grams
    b. Between 50 and 100 grams
    c. More than 100 grams
    d. No idea
33. How often is the litter changed?

a. Multiple times per day
b. Every day
c. Multiple times per week
d. Once per week
e. I do not have a litter

34. Does your cat sit frequently on your lap?
    a. Never
    b. Every day
    c. Several times per week
35. Where does your cat sleep?
    (Multiple answers possible)
    Check all that apply
    a. Kitchen
    b. Living room
    c. Bedroom
    d. Garage/basement/barn
    e. Other
36. Who is taking care of your cat when you are (temporarily) not around?
    (Multiple answers possible)
    Check all that apply
    a. Shelter or kennel
    b. Neighbours, friend or family
    c. Other

You and your pet

37. Are you the main carer of the pet? Yes/No
    The main carer is the person that devotes most time to the pet (walking the dog, feeding the cat, etc.)
38. Do you have other pets? Yes/No
39. How many years do you own your pet?
    a. Less than 1 year
    b. 1 to 5 years
    c. 5 to 10 years
    d. More than 10 years
40. Why did you get your pet?
    (Multiple answers possible)
    Check all that apply
    a. For myself
    b. For the kids
    c. For my job
    d. Other

Interactions between you and your pet

41. How do you and your pet go along?
    Based on The Pet Bonding Scale (Angle, 1994)
    Mark only one oval per row.

	Strongly agree	Agree	Neutral	Disagree	Strongly disagree
My pet can make me laugh	◯	◯	◯	◯	◯
I have a lot of fun with my pet	◯	◯	◯	◯	◯

My pet makes me feel important	◯	◯	◯	◯	◯
I have warm feelings when I think about my pet	◯	◯	◯	◯	◯
I like to spend a lot of time with my pet	◯	◯	◯	◯	◯
My pet loves me	◯	◯	◯	◯	◯
My pet misses me when I am gone	◯	◯	◯	◯	◯
I like to talk to my pet about things that are important to me	◯	◯	◯	◯	◯
I like to talk to my pet	◯	◯	◯	◯	◯
My pet understands my feelings	◯	◯	◯	◯	◯
I can tell secrets to my pet	◯	◯	◯	◯	◯
Sometimes my only friend is my pet	◯	◯	◯	◯	◯
My pet loves me no matter what	◯	◯	◯	◯	◯

42. How do you and your pet go along?
Based on The Pet Bonding Scale (Angle, 1994)
Mark only one oval per row.

	Strongly agree	Agree	Neutral	Disagree	Strongly disagree
One of my favourite things to do is to spend time with my pet	◯	◯	◯	◯	◯
My pet is an important part of my family	◯	◯	◯	◯	◯
My pet understands what I say	◯	◯	◯	◯	◯
I would be very upset if something happened to my pet	◯	◯	◯	◯	◯
I try to protect my pet	◯	◯	◯	◯	◯
I keep pictures of my pet	◯	◯	◯	◯	◯
My pet stays close to me when I am upset	◯	◯	◯	◯	◯
My pet has feelings	◯	◯	◯	◯	◯
I think about my pet when we are not together	◯	◯	◯	◯	◯
I miss my pet when I am not around	◯	◯	◯	◯	◯
My pet is important to me	◯	◯	◯	◯	◯
I am proud of my pet	◯	◯	◯	◯	◯

43. Does your pet communicate with you?
    Mark only one oval per row.

	Never	Sometimes	Often
By meowing or barking	◯	◯	◯
By means of body language (posture)	◯	◯	◯
By touching you (with head/legs)	◯	◯	◯
By scratching (against a door, for example)	◯	◯	◯
By looking at you	◯	◯	◯
By sniffing/recognizing your smell	◯	◯	◯

44. Do you like watching your pet? Yes/No
45. Do you like touching your pet? Yes/No
46. I consider my relationship with my pet to be: Bad/Normal/Good
47. Can you tell your pet is ill by its smell? Yes/No/Don't know
48. Do you sometimes attribute human characteristics to your pet? Yes/No
49. Do you think you look like your pet?
    (Multiple answers possible)
    Check all that apply.
    a. In behaviour
    b. In appearance
    c. Other
    d. No
50. Do you like taking care of your pet? Yes/No

Your pet and its emotions

51. Did you ever see these emotions expressed by your pet?
    Mark only one oval per row.

	Never	Sometimes	Often	No idea
Anger	◯	◯	◯	◯
Joy	◯	◯	◯	◯
Sadness	◯	◯	◯	◯
Disgust	◯	◯	◯	◯
Fear	◯	◯	◯	◯
Surprise	◯	◯	◯	◯
Shame	◯	◯	◯	◯
Jealousy	◯	◯	◯	◯
Disappointment	◯	◯	◯	◯

Compassion				

52. Do you think that these emotions are caused by the behaviour of yourself (or your housemates), in other words, do you directly affect the behaviour of your pet?
    Mark only one oval per row.

	Never	Sometimes	Often	No idea
Anger				
Joy				
Sadness				
Disgust				
Fear				
Surprise				
Shame				
Jealousy				
Disappointment				
Compassion				

53. Can you describe in a few words/phases of your pet's character?

____

____

____

____
